# GINS2 is a novel prognostic biomarker and promotes tumor progression in early-stage cervical cancer

**DOI:** 10.3892/or.2021.8016

**Published:** 2021-03-16

**Authors:** Fei Ouyang, Junling Liu, Meng Xia, Chuyong Lin, Xianqiu Wu, Liping Ye, Libing Song, Jun Li, Jing Wang, Peng Guo, Mian He

Oncol Rep 37: 2652-2662, 2017; DOI: 10.3892/or.2017.5573

Subsequently to the publication of the above article, the authors have realized that Figs. 2 and 6 each contained an incorrectly assembled data panel: Essentially, a couple of the data panels had been inadvertently selected twice for these figures.

The corrected versions of Figs 2 and 6, including the correct data for the H&E staining of the tissue of patient 4 in Fig. 2C and the invaded cells of RNAi#2 of the Siha cell line in Fig. 6C, are shown below and on the next page. All the authors agree to the publication of this Corrigendum, and they thank the Editor of *Oncology Reports* for allowing them the opportunity to publish it. Furthermore, they apologize to the readership for any inconvenience caused.

## Figures and Tables

**Figure 2.**
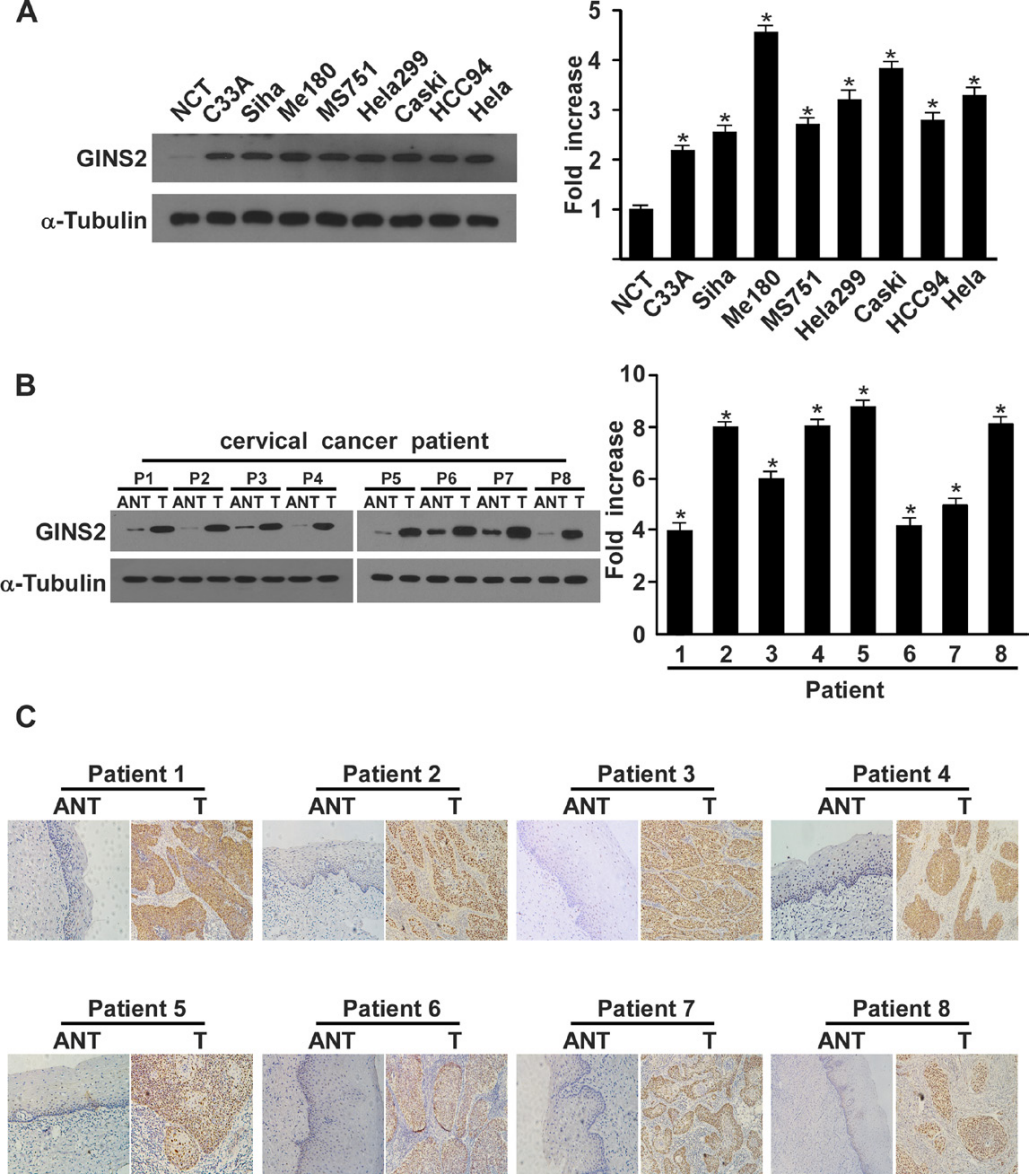
Western blotting, qPCR, and IHC determination of GINS2 mRNA and protein expression. (A) Western blotting and qPCR of GINS2 expression in normal cervical tissues and cervical cancer cell lines. (B) Western blotting of GINS2 expression in 8 pairs of matched cervical cancer (T) and adjacent non-cancerous cervical tissues (ANT) and average T/ANT ratios of GINS2 mRNA expression quantified by qPCR in 8 pairs of matched cervical cancer tissues. Expression levels were normalized to α-tubulin expression. Error bars represent standard deviation (SD) calculated from 3 parallel experiments. *P<0.05. (C) IHC assay of GINS2 protein expression in 8 pairs of matched cervical cancer tissues.

**Figure 6.**
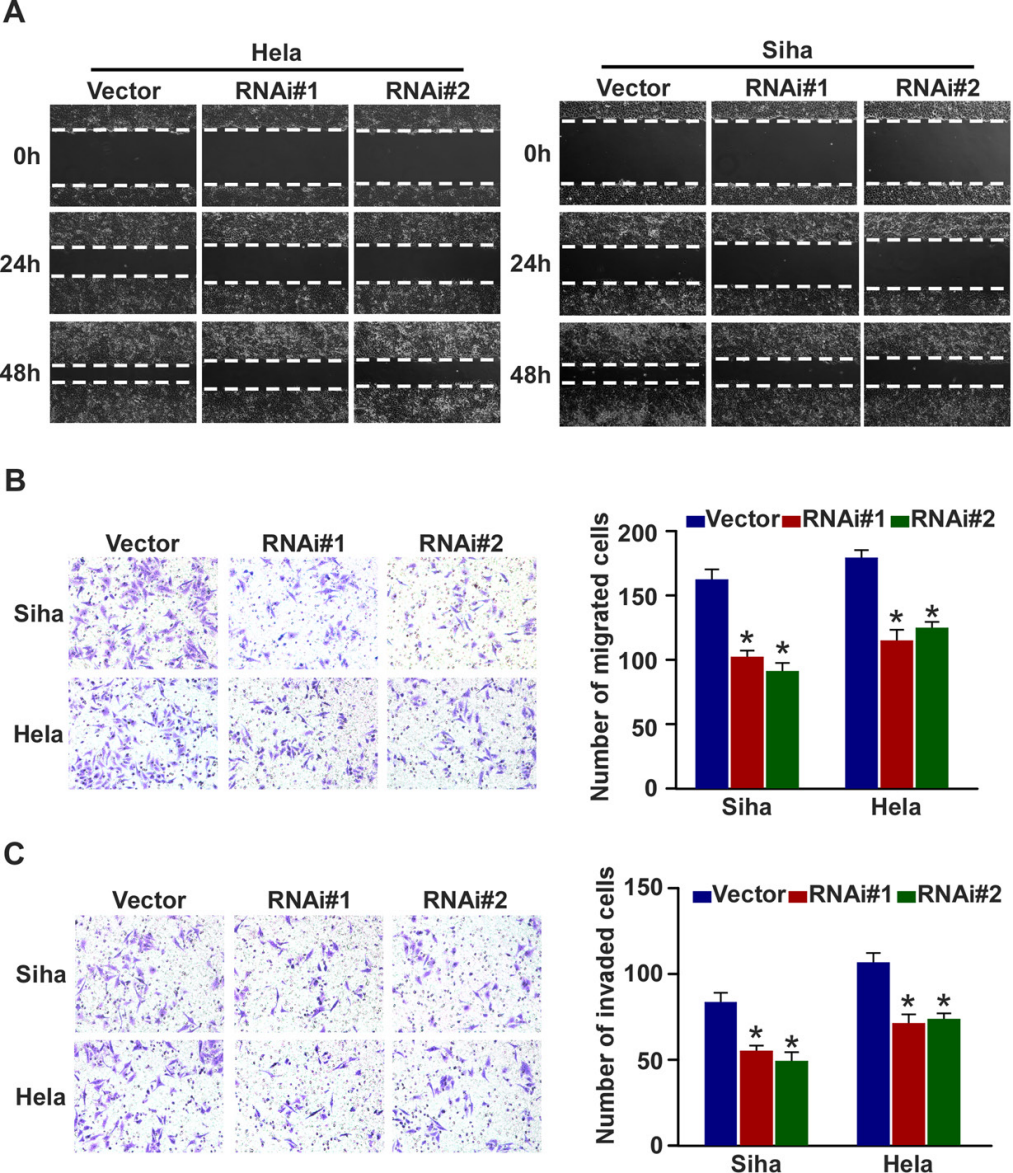
GINS2 is essential for cervical cancer cell migration and invasion. (A) Cell mobility was measured by examining the rate of wound closure at 0, 24 and 48 h. Original magnification, ×200. (B) Transwell migration assay investigation of the mobility properties induced by fetal bovine serum. Original magnification, ×200; *P<0.05. (C) Transwell invasion assay of the invasive properties induced by fetal bovine serum. Original magnification, ×200; *P<0.05. Bars represent the mean ± SD of 3 independent experiments

